# Chewing Ability and the Quality of Life: A Cross-Sectional Study to Assess the Relationship Between Tooth Wear and Oral Health

**DOI:** 10.7759/cureus.41906

**Published:** 2023-07-14

**Authors:** Rangoli Srivastava, Pradeep Tangade, Vikas Singh, Surbhi Priyadarshi, Sasmita Dalai, Priya Agarahari, Sonal Subhangi, Tannu Kumari, Ashutosh K Singh, Prabhat K Singh

**Affiliations:** 1 Department of Public Health Dentistry, Teerthanker Mahaveer Dental College and Research Centre, Moradabad, IND; 2 Department of Public Health Dentistry, SGT University (Shree Guru Gobind Singh Tricentenary University) Faculty of Dental Sciences, Gurugram, IND; 3 Department of Public Health Dentistry, Adesh Institute of Dental Sciences and Research, Bathinda, IND

**Keywords:** tooth wear, oral health-related quality of life, functional limitation, quality of life, abfraction, erosion, attrition, abrasion

## Abstract

Introduction: It is important to understand how a very common prevalent condition of tooth wear (TW) impacts a person’s day-to-day oral health. An emerging concept of measuring the parameter of oral health-related quality of life (OHRQoL), which evidently impacts the daily living of a person, makes it practical to examine the correlation between TW and OHRQoL. For measuring the OHRQoL, we can apply various methods, and the most effective is the use of the Oral Health Impact Profile (OHIP) questionnaire. Accordingly, the aim of this study was to assess the correlation between TW and the OHRQoL among adult patients attending a dental college and hospital.

Methods: A cross-sectional research was performed on patients who visited the outpatient department of Teerthanker Mahaveer Dental College and Research Centre, Moradabad, India. Initially, the sociodemographic details of patients, including their oral hygiene and dietary habits, were recorded. This step was followed by the assessment of TW using the Smith and Knight TW index. Then, the translated and validated version of the OHIP questionnaire was filled up, in which the patients were asked to rate each question on a Likert scale, with five points ranging from 0 to 4, where 0 = never, 1 = hardly ever, 2 = occasionally, 3 = fairly often, and 4 = very often.

Results: Based on a clinical examination on 630 subjects and the OHIP questionnaire responses from the participants, a significantly remarkable association (p ≤ 0.05) was found using a chi-square test between TW and the OHRQoL. In particular, TW was linked to other sociodemographic data and various lifestyle, dietary, and drinking habits. Along with the OHRQoL, TW also showed a positive correlation with gender. Using the chi-square test, a statistically significant association between age and TW was observed, with p-value = 0.004. Meanwhile, the place of residence did not show any association with TW. Educational qualifications of patients, visits to dental clinics, and reasons for dental visits showed very significant association with TW. Oral hygiene aids, materials used, frequency of brushing, and brushing technique did not have any association with TW as per the results obtained. A highly significant association was found between consumption of fruit drinks, citric drinks, and beverages and TW in the adult patients. Among all the domains of the OHIP questionnaire, the physical pain domain was the most affected, followed by the physical disability domain.

Conclusion: We conclude that TW has a direct association and positive correlation with the OHRQoL. As TW was increasing, so were the OHIP values, which indicated a lesser OHRQoL. The study also presents information on how to maintain a regular and healthy dietary lifestyle and oral hygiene to combat the impacts of TW.

## Introduction

Tooth wear (TW) and oral health are two interconnected aspects that significantly impact an individual's overall quality of life (QoL). Chewing is an essential function for proper nutrition and digestion, and any disturbances in this process can lead to various oral health issues and compromised well-being. The wearing down of teeth, known as dental attrition, can occur due to several factors, including bruxism (teeth grinding), malocclusion (misalignment of teeth), and certain dietary habits. Dental attrition can lead to the loss of tooth structure, altered occlusion (bite alignment), and heightened tooth sensitivity, among other concerns. These issues can impact an individual's ability to bite, chew, and speak comfortably, potentially leading to difficulty in consuming a balanced diet and affecting overall nutrition [1,2].

Furthermore, dental attrition and related oral health problems can have broader consequences beyond immediate physical discomfort. Psychological well-being may be affected, as individuals may feel self-conscious about their dental appearance and experience a decline in self-esteem. In addition, chronic pain associated with dental attrition can negatively impact daily activities and quality of sleep, leading to decreased overall QoL [1,2].

Understanding the relationship between TW and oral health is crucial for implementing effective preventive measures and developing appropriate treatment strategies. Indices are the most accurate approach to monitor deviation among teeth in massive populations. Regardless of the etiology, Smith and Knight developed the broader idea of evaluating the wear that has occurred on a tooth per surface [3]. If prevalent at above-normal levels, TW, a biological phenomenon in and of itself, could have a crippling impact on the oral health-related quality of life (OHRQoL). The extent until any individual appreciates the significant likelihood of having the benefit of life is what is meant by “QoL” [4].

The term OHRQOL refers to one's perspective as to how one's dental health affects every aspect of one's life and general health. The most commonly utilized tool for assessing dental health and its effects on people's QoL is the Oral Health Impact Profile (OHIP). Slade created a 14-item questionnaire variant of OHIP-49 because the initial OHIP had 49 questions and was built on a conceptual perspective created by the WHO, which was modified for dental health by Locker [4]. A 14-item checklist with self-reported functional impairment, pain, and impairment due to dental conditions is used to quantify these factors. Accordingly, in this study, we assessed the correlation between TW and the OHRQoL among adult patients attending a dental college and hospital [4,5].

## Materials and methods

A cross-sectional study was conducted on nearly 630 patients attending the outpatient department of Teerthanker Mahaveer Dental College and Research Centre, Moradabad, India, over a duration of six months commencing from January 2021 to July 2021, to determine the amount of TW present in them and its impact on the OHRQoL.

The sample size was estimated on the basis of the prevalence of a pilot study conducted on 40 participants: N = Z2PQ/ L2, N = sample size, Z = point of normal distribution (as per the table under the normal curve for the given confidence level of 95%) = 1.96, P= proportion or prevalence of interest = 37.97%, Q = 1-p (alternate prevalence) = 62.03, and L = 10% of prevalence = 3.8.

N = (1.96)2 37.97×62.0/ (3.8)2 = 624

Ethical clearance was obtained from the ethical committee of Teerthanker Mahaveer Dental College and Research Centre with institutional review board number TMDCRC/IEC/20-21/PHD2.

Questionnaires that were used had a content validity index of 0.95 and Cronbach’s alpha value of 0.85, which was validated by subject experts. The sociodemographic details of the participants were noted, and they were asked to fill out the OHIP-14 questionnaire for the assessment of the OHRQoL. Socioeconomic status was classified according to the modified BG Prasad socioeconomic classification (updated 2020). Annual income was then obtained from the subjects and divided by 12 (months), and then the value was divided by the number of family members to get the per capita income of the family. The per capita income was subjected to the modified Prasad’s classification.

By doing the test, the examiners were educated beforehand, and calibration was already done on the predetermined individuals twice, with half an hour gap between each. The examiner was accompanied by one recording clerk, who was also a dentist and was well versed with the indices used and coding systems, to note down the information collected during the examination. The recording clerk was calibrated and standardized with the scoring criteria and coding system of the research-based indices. TW was noted clinically with the help of Smith and Knight TW index. The recording of every particular surface was conducted separately. A score of 0 is allotted when there is zero deterioration or no change on any surface. A score of 1 means changes in the shape’s outermost layer. A score of 2 represents more than 30% of alteration on surfaces. A score of 3 is allotted when the dentine layer is exposed and when there is a defect of 1-2 mm in depth. A score of 4 is allotted when there is a defect more than 2 mm in depth along with pulp exposure [3].

Different techniques were utilized to assess the influence of alterations in the OHRQoL on dental-related results. The most popular technique for determining OHRQoL parameters is OHIP-14 [4]. It illustrates how dental diseases have an effect on an individual's overall health. The values of this scale range from 0 to 56 for the 14 questions. In our study, we used a translated version of OHIP-14 [5]. It is a self-reported questionnaire with an emphasis on seven impact factors or domains.

These domains individually describe the limitations that a person faces function-wise, the pain that a person bears physically, the discomfort that a person faces psychologically, the disability that a person faces physically, the disability that a person has psychologically, the disability that a person faces socially, and the conditions related to the feeling of handicappedness in a person.

The inclusion criteria included patients aged 35-44 years attending the outpatient department of Teerthanker Mahaveer Dental College and Research Centre. The exclusion criteria included adult patients with apparent medical/oral conditions (excluding TW), adult individuals who declined to take part in the research, adult patients who are suffering from any systemic disease, and adult patients who are undergoing orthodontic treatments.

Clinical examination and questionnaire filling regarding their sociodemographic details and the OHIP-14 questionnaire were performed. The OHIP-14 questionnaire was a translated and validated Hindi version. For testing the validity of the OHIP-14 questionnaire in our population, we took a sample size of 40 participants for our pilot study. The Cronbach's alpha value of our pilot study was 0.85, which showed a good internal consistency. For the convenience of patients, an already Hindi-translated version of OHIP-14 was used. With the aid of the backtranslation method to equalize the social aspect, OHIP-14 was modified in both ways (as per language and as per ethnicity). The differences between the translated version and original version of OHIP were further checked and evaluated, thus proving the point that both versions are more or less the same. The rechecked version of the OHIP-14 (Hindi language) is appropriate to be taken for any pilot study. Nearly 7-11 minutes were taken by the participants to fill the questionnaire. After recording the sociodemographic details of the patients, their oral cavity, particularly regarding TW, was assessed clinically, and based on the Smith and Knight criteria of TW, values of 0, 1, 2, and 3 were given for each tooth surface as per the amount of TW present in them. After the clinical assessment, the patients were asked to mark each question of the OHIP-14 on an assessment sheet as per the impact of TW on their oral health on a five-point likert scale, where 0 = never, 1= hardly ever, 2 = occasionally, 3 = fairly often, and 4 = very often.

Data were analyzed through a descriptive and inferential analysis using IBM SPSS Statistics for Windows, Version 24 (Released 2016; IBM Corp., Armonk, New York, United States). With the help of descriptive statistics, details were described. A chi-square analysis was done to determine the association between TW and other sociodemographic factors using OHIP-14. Analysis of variance (ANOVA) was done to assess the mean impact of OHIP-14 domain scores on TW. The value of significance was kept at 5%.

The Shapiro-Wilk test was used to test the normality of data, and for each parametric data, the value for each set of data ensures that the data distribution is normal (Table 1).

**Table 1:** Normality test of the data df: degrees of freedom; OHIP-14: Oral Health Impact Profile-14; Sig.: significance

Parameter	Shapiro-Wilk test values		
	Statistics	df	Sig.
Age	0.958	111	0.001
Tooth wear	0.939	111	0.000
OHIP-14	0.967	111	0.008

## Results

Descriptive statistics were employed for the calculation of descriptive details. A chi-square test was used to assess the association between gender and TW, age and TW, socioeconomic status and TW, place of residence and TW, educational qualification and TW, last dental visit and TW, reasons for dental visit and TW, oral hygiene used and TW, oral hygiene material used and TW, frequency of brushing and TW, consumption of fruit/citric acids and TW, consumption of beverages/carbonated drinks and TW, and brushing technique and TW (Table 2).

**Table 2:** Descriptive statistics

Subjects	Frequency	Percent
Gender		
Male	343	54.4
Female	287	45.6
Age		
35	52	8.30%
36	60	9.50%
37	69	11%
38	103	16.30%
39	62	9.80%
40	86	13.70%
41	49	7.80%
42	70	11.10%
43	39	6.20%
44	34	5.40%
45	6	1%
Socioeconomic status		
Upper class	42	6.70%
Upper middle class	88	14%
Lower middle class	222	35.20%
Upper lower class	205	32.50%
Lower class	73	11.60%
Place of residence		
Village	159	25.20%
City	471	74.80%
Educational qualifications		
10^th^ pass	57	9%
12^th^ pass	144	22.90%
Graduation	384	61%
Post-graduation	45	7.10%
Dental visits		
Last 6 months	42	6.70%
Last 6 months-1 year	82	13%
1-2 years	200	31.70%
More than 2 years	149	23.70%
None	157	24.90%
Reason for dental visits		
General check-up	101	16%
Specific treatment	371	58.90%
None	158	25.10%
Oral hygiene aids used		
Toothbrush	417	66.20%
Finger	196	31.10%
Tree-stick	17	2.70%
Oral hygiene material used		
Toothpaste	363	57.60%
Toothpowder	250	39.70%
Charcoal	11	1.70%
Salt	6	1%
Frequency of brushing		
Once	387	61.40%
Twice	178	28.30%
Thrice	65	10.30%
Consumption of fruit/citric drinks		
Yes	360	57.10%
No	270	42.90%
Consumption of beverages/carbonated drinks		
Yes	302	47.90%
No	328	52.10%
Brushing technique		
Horizontal	392	62.20%
Vertical	164	26%
Combination	74	11.70%

**Figure 1:**
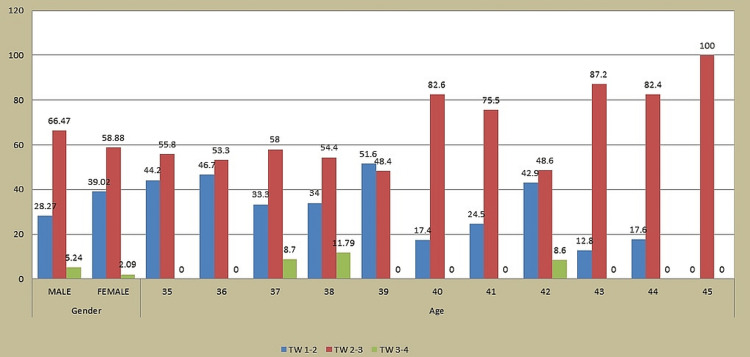
Association of gender and age with tooth wear (TW)

There were nearly 343 (54.4%) males and 287(45.6%) females aged 35-44 years. A statistically significant relationship was discovered between gender and TW (p-value = 0.004) using the chi-square test. The mean age of participants was 39.2±2.623. A statistically significant relationship was also discovered between age and TW (p-value = 0.004) using the chi-square test (Figure 1).

**Figure 2:**
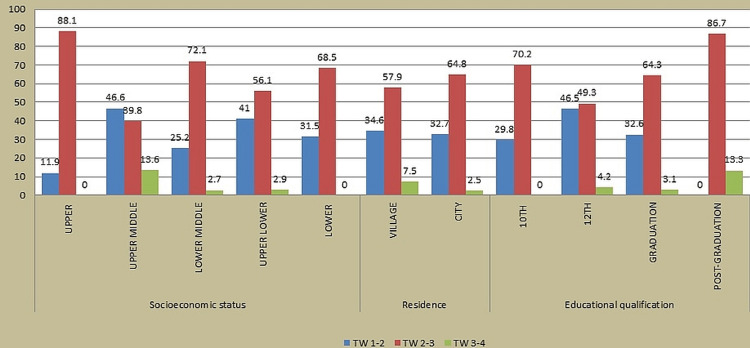
Association of socioeconomic status, residence, and educational qualification with tooth wear (TW)

After classifying the study participants as per socioeconomic status, majority were found to be from the upper middle class, followed by the lower middle class. A statistically significant relationship was discovered between socioeconomic status and TW (p value = 0.0042) using the chi-square test. Majority (74.8%) of the participants were inhabitants of a city, and a statistically insignificant relationship was found between the residence of a person and TW (p value = 0.012) using the chi-square test. Majority (61%) of the participants were 12th pass students, and a statistically significant relationship was discovered between the educational status of a person and TW (p value < 0.001) using the chi-square test (Figure 2).

**Figure 3:**
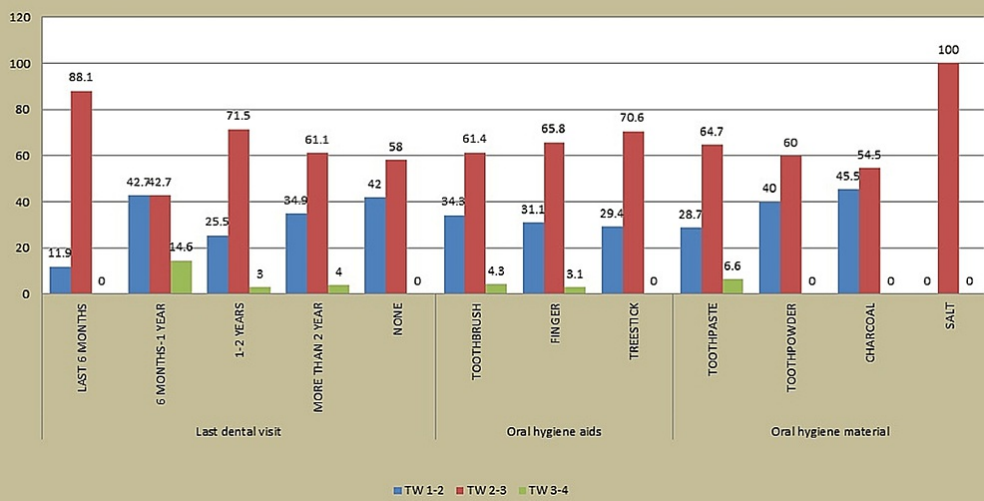
Association of last dental visit and oral hygiene aids and material with tooth wear (TW)

Majority (31.7%) of the participants had a dental visit in the past 1-2 years, and a statistically significant relationship was discovered between last dental visit and TW using the chi-square test. Majority (58.9%) of the participants visited a dentist for some specific treatments, and a statistically significant relationship was discovered between reason of dental visit and TW (p value < 0.001) using the chi-square test. Majority (66.2%) of the participants used toothbrush as an oral hygiene aid, and a nonsignificant relationship was discovered between aids of oral hygiene used and TW (p value = 0.686). Majority (57.6%) of the participants used toothpaste as an oral hygiene material, and a statistically significant relationship was discovered between the material of oral hygiene used and TW (p value = 0.001) using the chi-square test (Figure 3).

**Figure 4:**
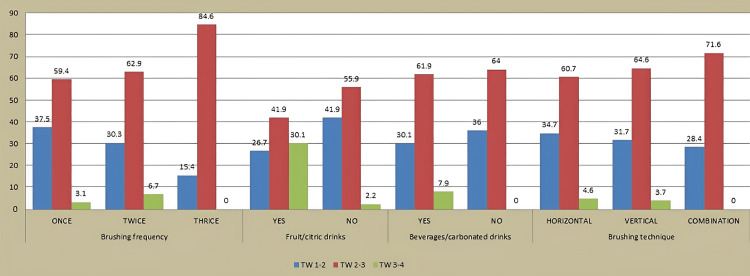
Association of brushing frequency, drinking habits, and brushing technique with tooth wear (TW)

Majority (61.4%) of the participants brushed their teeth only once, and a statistically significant relationship was discovered between the brushing frequency of a person and TW (p value < 0.001) using the chi-square test. A highly significant association was observed between consumption of fruit drinks, citric drinks, and beverages and TW among adults (Figure 4).

A post-hoc test was used to assess the intergroup comparison of the range of TW (mild, moderate, and severe) with the OHIP range (no impact, mild, moderate, and severe). Among the participants who had TW ranging between 1 and 2, majority (161, 77%) had mild impact on the OHRQoL, followed by 45 (21.5%) having no impact on the OHRQoL, two (1%) having moderate impact on the OHRQoL, and one (0.5%) having severe impact on the OHRQoL. Among the participants who had TW ranging between 2 and 3, majority (92.4%) had mild impact on the OHRQoL, followed by 23 (5.8%) having no impact on the OHRQoL, six (1.5%) having moderate impact on the OHRQoL, and one (0.3%) having severe impact on the OHRQoL. Amongst the participants who had TW ranging between 3 and 4, majority (87.5%) had mild impact on the OHRQoL, followed by two (8.3%) having moderate impact on the OHRQoL and one (4.2%) having severe impact on the OHRQoL. As regards the overall TW, 549 (87.1%) participants had mild impact on the OHRQoL, followed by 68 (10.8%) having no impact on the OHRQoL, 10 (1.6%) having moderate impact on the OHRQoL, and three (0.5%) having severe impact on the OHRQoL. Thus, a statistically significant relationship was discovered between TW and the OHRQoL when the value of significance was 0.001 (Table 3).

**Table 3:** Association between TW and OHRQoL TW: tooth wear; OHRQoL: oral health-related quality of life

Tooth wear (TW)	OHIP				Chi-square value	p-value
	No impact range (from zero till 14)	Mild range (from 15 till 28)	Moderate range (from 29 till 32)	Severe range ( from 33 till 56)	52.545	0.001
TW 1-2	45 (21.5%)	161 (77%)	2 (1%)	1 (0.5%)		
TW 2-3	23 (5.8%)	367 (92.4%)	6 (1.5%)	1 (0.3%)		
TW 3-4	0 (0%)	21 (87.5%)	2 (8.3%)	1 (4.2%)		
Total	68 (10.8%)	549 (87.1%)	10 (1.6%)	3 (0.5%)		

ANOVA test was used to assess the mean OHIP-14 domain scores’ impact on the severity of TW among the studied population. In addition, among all the domains of OHIP-14, physical pain was the most affected domain, followed by physical disability. A highly and statistically significant relationship was discovered between TW and all domains of OHIP-14 translated version when the value of significance was 0.001 (Table 4).

**Table 4:** Mean OHIP-14 domain scores and its effect with relation to severity of tooth wear (TW) among the participants OHIP: Oral Health Impact Profile; TW: tooth wear

TW severity	Functional limitation	Physical pain	Psychological discomfort	Physical disability	Psychological disability	Social disability	Handicap	Overall OHIP
1-2	2.51±0.659	2.56±0.913	2.45±0.671	2.48±0.872	2.3±0.67	2.41±0.66	2.46±0.63	17.17±4.005
2-3	2.76±0.68	3.06±0.944	2.94±0.716	3.02±0.738	2.7±0.75	2.75±0.74	2.84±0.74	20.09±3.84
3-4	3.38±0.97	3.71±0.908	3.5±1.06	3.38±1.173	3.13±1.3	3.33±1.09	3±1.3	23.42±7.16
Overall score	2.7±0.7	2.92±0.972	2.8±0.76	2.86±0.84	2.59±0.79	2.66±0.76	2.72±0.76	19.25±4.36
p value	0.001	0.001	0.001	0.001	0.001	0.001	0.001	0.001

## Discussion

TW is becoming a very common problem among the younger and older adults worldwide. Its etiology is multifactorial, so we cannot designate one single factor for its etiology. As per some studies, TW is caused by the habits of a person, whereas some said that TW’s etiology is designated to the diet type of a person. In our study, we correlated TW with the OHRQoL, and a positive correlation was found between TW and the OHRQoL. Based on a study conducted by Smith et al. [6], in which they assessed TW and its etiological factors, the toothbrushing frequency showed a positive correlation with TW. This finding implies that inadequate or excessive toothbrushing practices may contribute to TW, and inadequate or aggressive toothbrushing techniques, especially with a hard-bristled toothbrush, can wear down the enamel and expose the underlying dentin layer, emphasizing the importance of maintaining proper oral hygiene routines. 

Based on a study conducted by Al Zarea et al. [7] on TW, the most common risk factors associated with TW are acidic food and drinks, which is also in line with the inference of our cross-sectional study. Consuming acidic beverages regularly may erode tooth structure and contribute to TW. Based on a study conducted by Barlett et al. [8] on TW and associated risk factors, a nonsignificant association between TW and the frequency of intake of carbonated drinks exists. As per a study conducted by Khalifa et al. [9] among adults and their TW, it was assessed that the gender of that population and their socioeconomic status showed a positive correlation with TW.

Furthermore, in a study conducted by Barlett et al. [10] on TW, they concluded that erosion was directly associated with age and gender showed no association with erosive wear. As per a study conducted by Allaq et al. [11] regarding TW in a population, TW is directly linked to the age of that population, which is also in line with our study’s inference. In addition, factors, such as changes in saliva composition and oral health conditions, which become more common with age, can contribute to TW. As per a study conducted by Kumar S et al. [12] on assessing TW, it was concluded that the residence location of people, either rural or urban, is not associated with TW, which is the same as the inference of our study conducted on adults in the age group 35-44 years.

As per a study conducted by Praveena J et al. [13] regarding TW in a population and its association with the OHRQoL, with the help of the OHIP, it was assessed that all OHIP domains were significantly associated with TW except the functional limitation domain. As per a study conducted by Mehta SB et al. [14] among adults who were assessed for their dental erosion using a basic erosive wear examination and its association with the OHIP, it was concluded that as the assessed score was increased, so was their OHIP score; hence, it showed a positive correlation with it. As per a study conducted by Patel J et al. [15] among adults, TW showed an indirect association with the OHRQoL. However, those who had moderate and severe TW showed a positive association with the OHRQoL. As per a study conducted by Kannan A et al. [16] regarding TW, it was assessed that TW was directly associated with the consumption of acidic beverages and those who consumed these beverages on a regular basis had lower OHRQoL. As per a study conducted by Papagianni CE et al. [17] on TW, the results concluded that TW was directly linked with the OHRQoL.

In our study, the measure of the OHRQoL depicts how one’s oral conditions impacts the total well-being of an individual and further the QoL. Research in the last previous years has taken into consideration various diseases and conditions and their impacts on the OHRQoL, and so was the purpose of our study too. In the previous studies, the main oral health conditions that were taken into consideration were edentulousness, tooth caries, and several periodontal diseases. Meanwhile, in our study, we relevantly took the condition of TW into consideration. There were many limited studies done with reference to finding the impact of TW on the OHRQoL. This study is one of the first few studies done to assess the impact of TW on the OHRQoL with the help of various parameters in India. As the cases of TW seem to rise, such a study would be beneficial for us to understand and eliminate possible factors that lead to poor OHRQoL.

Assessing more than 600 patients with TW in a shorter duration of time in a specific population justifies the alarming increase in the amount of TW in patients over the years. It was difficult to assess the etiology of TW, and possible etiology as per the factors assessed could be brushing pattern and liquid intake in one’s diet.

The study has various limitations. A single-center study was conducted, which limits the generalizability of the findings to a broader population. Hence, the study may not represent the entire population or different geographical areas. The study utilized a cross-sectional design, which captures data at a specific point in time. Longitudinal studies would provide more robust evidence for understanding the progression and impact of TW over time. The data collection involved self-reporting by patients regarding their oral hygiene and dietary habits. Self-reported data may be subject to recall bias or social desirability bias, potentially affecting the accuracy and reliability of the information gathered. The study included a sample of 630 subjects from the outpatient department, which may not be representative of the larger population. In addition, there might be selection bias present if the sample is not randomly selected, potentially limiting the generalizability of the results. The study did not include a control group for comparison, which limits the ability to draw definitive conclusions about the specific impact of TW on the OHRQoL. Our study included predominantly urban participants, and hence socioeconomic status can be a possible confounding factor for the QoL. The possible etiology and type of TW and its impact on the OHRQoL will lead to a better understanding of the scope of this condition and what future preventive measures can be taken to have a better oral health.

## Conclusions

The study did not find any association between TW and the place of residence or oral hygiene practices, such as the use of oral hygiene aids, brushing frequency, and brushing technique. However, the consumption of fruit drinks, citric drinks, and beverages showed a highly significant association with TW among adults. Assessing the OHRQoL using the OHIP questionnaire revealed that TW had a direct association and a positive correlation with the OHRQoL. The domains most affected by TW were physical pain and physical disability.

These findings emphasize the importance of maintaining a regular and healthy dietary lifestyle, along with good oral hygiene practices, in order to prevent or minimize the impact of TW. TW cannot be reversed, but its further degradation can be prevented by monitoring the etiological causes.

## References

[1] Lee A, He LH, Lyons K, Swain MV (2012). Tooth wear and wear investigations in dentistry. J Oral Rehabil.

[2] López-Frías FJ, Castellanos-Cosano L, Martín-González J, Llamas-Carreras JM, Segura-Egea JJ (2012). Clinical measurement of tooth wear: tooth wear indices. J Clin Exp Dent.

[3] Smith BG, Knight JK (1984). An index for measuring the wear of teeth. Br Dent J.

[4] Slade GD (1997). Derivation and validation of a short-form oral health impact profile. Community Dent Oral Epidemiol.

[5] Deshpande NC, Nawathe AA (2015). Translation and validation of Hindi version of Oral Health Impact Profile-14. J Indian Soc Periodontol.

[6] Smith WA, Marchan S, Rafeek RN (2008). The prevalence and severity of non-carious cervical lesions in a group of patients attending a university hospital in Trinidad. J Oral Rehabil.

[7] Al-Zarea BK (2012). Tooth surface loss and associated risk factors in northern saudi arabia. ISRN Dent.

[8] Bartlett DW, Fares J, Shirodaria S, Chiu K, Ahmad N, Sherriff M (2011). The association of tooth wear, diet and dietary habits in adults aged 18-30 years old. J Dent.

[9] Al-Khalifa KS (2020). The prevalence of tooth wear in an adult population from the eastern province of Saudi Arabia. Clin Cosmet Investig Dent.

[10] Bartlett DW, Lussi A, West NX, Bouchard P, Sanz M, Bourgeois D (2013). Prevalence of tooth wear on buccal and lingual surfaces and possible risk factors in young European adults. J Dent.

[11] Al-Allaq T, Feng C, Saunders RH (2018). Anterior tooth wear and quality of life in a nursing home population. Spec Care Dentist.

[12] Kumar A, Puranik MP, Sowmya KR, Rajput S (2019). Impact of occupational dental erosion on oral health-related quality of life among battery factory workers in Bengaluru, India. Dent Res J (Isfahan).

[13] Praveena J, Battur H, Fareed N, Khanagar S. (2018). The prevalence and impact of teeth wear on oral health related quality of life among rural adult population of Sullia taluk, D.K. J Indian Assoc Public Health Dent.

[14] Mehta SB, Loomans BA, Banerji S, Bronkhorst EM, Bartlett D (2020). An investigation into the impact of tooth wear on the oral health related quality of life amongst adult dental patients in the United Kingdom, Malta and Australia. J Dent.

[15] Patel J, Baker SR (2020). Is toothwear associated with oral health related quality of life in adults in the UK?. Community Dent Health.

[16] Kanaan M, Brabant A, Eckert GJ, Hara AT, Carvalho JC (2022). Tooth wear and oral-health-related quality of life in dentate adults. J Dent.

[17] Papagianni CE, van der Meulen MJ, Naeije M, Lobbezoo F (2013). Oral health-related quality of life in patients with tooth wear. J Oral Rehabil.

